# 2,3-Dibromo-3-phenyl-1-(3-phenyl­sydnon-4-yl)propan-1-one

**DOI:** 10.1107/S1600536811008026

**Published:** 2011-03-09

**Authors:** Hoong-Kun Fun, Madhukar Hemamalini, Nithinchandra, Balakrishna Kalluraya

**Affiliations:** a X-ray Crystallography Unit, School of Physics, Universiti Sains Malaysia, 11800 USM, Penang, Malaysia; b Department of Studies in Chemistry, Mangalore University, Mangalagangotri, Mangalore 574 199, India

## Abstract

In the title compound [systematic name: 4-(2,3-dibromo-3-phenyl­propano­yl)-3-phenyl-1,2,3-oxadiazol-3-ylium-5-olate], C_17_H_12_Br_2_N_2_O_3_, the oxadiazole ring is essentially planar, with a maximum deviation of 0.001 (3) Å. The central oxadiazole ring makes dihedral angles of 73.3 (2) and 29.0 (2)° with the adjacent and remote phenyl rings, respectively. In the crystal, adjacent mol­ecules are connected by C—H⋯O hydrogen bonds, forming a supra­molecular chain along the *c* axis. There is an intra­molecular C—H⋯O hydrogen bond, which generates an *S*(6) ring motif.

## Related literature

For applications of sydnones, see: Rai *et al.* (2008 ▶); Jyothi *et al.* (2008 ▶). For details of chalcones, see: Rai *et al.* (2007 ▶).
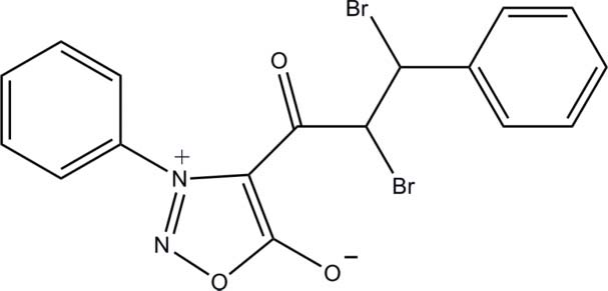


## Experimental

### 

#### Crystal data

C_17_H_12_Br_2_N_2_O_3_
*M* *_r_* = 452.11
Monoclinic, 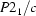

*a* = 11.9109 (3) Å
*b* = 17.5018 (3) Å
*c* = 8.5365 (2) Å
β = 94.960 (1)°
*V* = 1772.87 (7) Å^3^
*Z* = 4
Mo *K*α radiation
μ = 4.59 mm^−1^
*T* = 296 K
0.30 × 0.20 × 0.04 mm

#### Data collection

Bruker SMART APEXII CCD area-detector diffractometer
Absorption correction: multi-scan (*SADABS*; Bruker, 2005 ▶) *T*_min_ = 0.341, *T*_max_ = 0.849
23853 measured reflections
4082 independent reflections
1912 reflections with *I* > 2σ(*I*)
*R*_int_ = 0.068

#### Refinement

*R*[*F* ^2^ > 2σ(*F* ^2^)] = 0.043
*wR*(*F* ^2^) = 0.104
*S* = 0.98
4082 reflections
217 parameters
H-atom parameters constrained
Δρ_max_ = 0.48 e Å^−3^
Δρ_min_ = −0.44 e Å^−3^

### 

Data collection: *APEX2* (Bruker, 2009 ▶); cell refinement: *SAINT* (Bruker, 2009 ▶); data reduction: *SAINT*; program(s) used to solve structure: *SHELXTL* (Sheldrick, 2008 ▶); program(s) used to refine structure: *SHELXTL*; molecular graphics: *SHELXTL*; software used to prepare material for publication: *SHELXTL* and *PLATON* (Spek, 2009 ▶).

## Supplementary Material

Crystal structure: contains datablocks global, I. DOI: 10.1107/S1600536811008026/is2684sup1.cif

Structure factors: contains datablocks I. DOI: 10.1107/S1600536811008026/is2684Isup2.hkl

Additional supplementary materials:  crystallographic information; 3D view; checkCIF report

## Figures and Tables

**Table 1:** Hydrogen-bond geometry (Å, °)

*D*—H⋯*A*	*D*—H	H⋯*A*	*D*⋯*A*	*D*—H⋯*A*
C10—H10*A*⋯O2	0.98	2.35	3.028 (6)	126
C13—H13*A*⋯O2^i^	0.93	2.49	3.312 (6)	147

Symmetry code: (i) 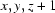.

**Fig. 1.**
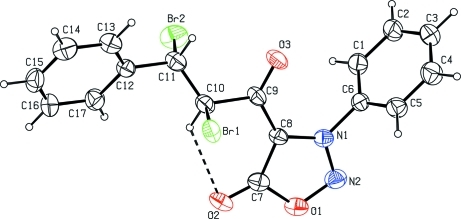
The asymmetric unit of the title compound, showing 30% probability displacement ellipsoids and the atom-numbering scheme. The intramolecular hydrogen bond is shown as a dashed line.

**Fig. 2.**
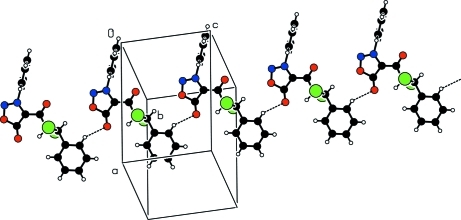
The crystal packing of the title compound.

## supplementary crystallographic information

### Comment

Sydnones constitute a well-defined class of mesoionic compounds that contain the 1,2,3-oxadiazole ring system. The study of sydnones still remains a field of interest because of their electronic structure and also because of the varied types of biological activities (Rai *et al.*, 2008). Recently, sydnone derivatives were found to exhibit promising anti-microbial properties (Jyothi *et al.*, 2008). Chalcones were obtained by the base-catalyzed condensation of 4-acetyl-3-aryl sydnones with aromatic aldehydes in alcoholic medium employing sodium hydroxide as catalyst at 0–5 °C. Bromination of chalcones with bromine in glacial acetic acid afforded dibromo chalcones (Rai *et al.*, 2007).

The molecular structure of the title compound is shown in Fig. 1. The oxadiazole (N1/N2/O1/C7/C8) ring is essentially planar, with a maximum deviation of 0.001 (3) Å for atom O1.  The central oxadiazole (N1/N2/O1/C7/C8) ring makes dihedral angles of 73.3 (2)° and 29.0 (2)° with the attached phenyl (C1–C6) and the terminal phenyl (C12–C17) rings, respectively.

In the crystal, (Fig. 2), the adjacent molecules are connected by intra and intermolecular C10—H10A···O2 and C13—H13A···O2 (Table 1) hydrogen bonds forming supramolecular chains along the *c*-axis. There is an intramolecular C—H···O hydrogen bond, which generates an *S*(6) ring motif.

### Experimental

1-(3^1^-Phenylsydnon-4^1^-yl)-3-(phenyl)-propen-1-one (0.01 mol) was dissolved in glacial acetic acid (2–30 ml) by gentle warming. A solution of bromine in glacial acetic acid (30% w/v) was added to it with constant stirring till the yellow color of the bromine persisted. The reaction mixture was stirred at room temperature for 1-2 hours. The solid which separated was filtered, washed with methanol and dried. It was then recrystallized from ethanol. Crystals suitable for X-ray analysis were obtained from 1:2 mixtures of DMF and ethanol by slow evaporation.

### Refinement

All H atoms were positioned geometrically (C—H = 0.93 Å) and were refined  using a riding model, with *U*_iso_(H) = 1.2*U*_eq_(C).

### Crystal data

**Table:**

C_17_H_12_Br_2_N_2_O_3_	*F*(000) = 888
*M**_r_* = 452.11	*D*_x_ = 1.694 Mg m^−^^3^
Monoclinic, *P*2_1_/*c*	Mo *K*α radiation, λ = 0.71073 Å
Hall symbol: -P 2ybc	Cell parameters from 3028 reflections
*a* = 11.9109 (3) Å	θ = 2.3–19.9°
*b* = 17.5018 (3) Å	µ = 4.59 mm^−^^1^
*c* = 8.5365 (2) Å	*T* = 296 K
β = 94.960 (1)°	Plate, colourless
*V* = 1772.87 (7) Å^3^	0.30 × 0.20 × 0.04 mm
*Z* = 4	

### Data collection

**Table:**

Bruker SMART APEXII CCD area-detector diffractometer	4082 independent reflections
Radiation source: fine-focus sealed tube	1912 reflections with *I* > 2σ(*I*)
graphite	*R*_int_ = 0.068
φ and ω scans	θ_max_ = 27.6°, θ_min_ = 1.7°
Absorption correction: multi-scan (*SADABS*; Bruker, 2005)	*h* = −15→15
*T*_min_ = 0.341, *T*_max_ = 0.849	*k* = −22→22
23853 measured reflections	*l* = −11→11

### Refinement

**Table:**

Refinement on *F*^2^	Primary atom site location: structure-invariant direct methods
Least-squares matrix: full	Secondary atom site location: difference Fourier map
*R*[*F*^2^ > 2σ(*F*^2^)] = 0.043	Hydrogen site location: inferred from neighbouring sites
*wR*(*F*^2^) = 0.104	H-atom parameters constrained
*S* = 0.98	*w* = 1/[σ^2^(*F*_o_^2^) + (0.0352*P*)^2^ + 0.6409*P*] where *P* = (*F*_o_^2^ + 2*F*_c_^2^)/3
4082 reflections	(Δ/σ)_max_ = 0.001
217 parameters	Δρ_max_ = 0.48 e Å^−^^3^
0 restraints	Δρ_min_ = −0.44 e Å^−^^3^

### Special details

**Table:**

Geometry. All esds (except the esd in the dihedral angle between two l.s. planes) are estimated using the full covariance matrix. The cell esds are taken into account individually in the estimation of esds in distances, angles and torsion angles; correlations between esds in cell parameters are only used when they are defined by crystal symmetry. An approximate (isotropic) treatment of cell esds is used for estimating esds involving l.s. planes.
Refinement. Refinement of F^2^ against ALL reflections. The weighted R-factor wR and goodness of fit S are based on F^2^, conventional R-factors R are based on F, with F set to zero for negative F^2^. The threshold expression of F^2^ > 2sigma(F^2^) is used only for calculating R-factors(gt) etc. and is not relevant to the choice of reflections for refinement. R-factors based on F^2^ are statistically about twice as large as those based on F, and R- factors based on ALL data will be even larger.

### Fractional atomic coordinates and isotropic or equivalent isotropic displacement parameters (Å^2^)

**Table:**

	*x*	*y*	*z*	*U*_iso_*/*U*_eq_	
Br1	0.16038 (4)	1.00790 (2)	0.96635 (6)	0.0826 (2)	
Br2	0.24594 (5)	0.75050 (3)	0.96598 (7)	0.1118 (3)	
O1	0.0435 (3)	0.92146 (17)	0.4180 (4)	0.0871 (9)	
O2	0.2052 (3)	0.94860 (17)	0.5666 (4)	0.0889 (10)	
O3	0.0007 (3)	0.8462 (2)	0.9185 (4)	0.0943 (10)	
N1	−0.0551 (3)	0.87006 (17)	0.5834 (5)	0.0648 (9)	
N2	−0.0581 (4)	0.8894 (2)	0.4355 (5)	0.0855 (11)	
C1	−0.1583 (4)	0.7596 (2)	0.6667 (6)	0.0827 (14)	
H1A	−0.0949	0.7295	0.6565	0.099*	
C2	−0.2551 (4)	0.7276 (3)	0.7140 (6)	0.0939 (16)	
H2A	−0.2574	0.6757	0.7365	0.113*	
C3	−0.3477 (5)	0.7719 (3)	0.7279 (6)	0.0957 (16)	
H3A	−0.4133	0.7502	0.7596	0.115*	
C4	−0.3441 (5)	0.8483 (3)	0.6951 (7)	0.1000 (16)	
H4A	−0.4076	0.8783	0.7048	0.120*	
C5	−0.2478 (4)	0.8815 (3)	0.6481 (6)	0.0827 (14)	
H5A	−0.2452	0.9334	0.6258	0.099*	
C6	−0.1562 (4)	0.8357 (2)	0.6350 (5)	0.0639 (11)	
C7	0.1121 (5)	0.9216 (2)	0.5614 (6)	0.0722 (12)	
C8	0.0427 (4)	0.8868 (2)	0.6687 (5)	0.0615 (11)	
C9	0.0669 (4)	0.8733 (2)	0.8348 (6)	0.0695 (12)	
C10	0.1842 (4)	0.8990 (2)	0.9026 (6)	0.0768 (13)	
H10A	0.2368	0.8972	0.8205	0.092*	
C11	0.2311 (4)	0.8574 (3)	1.0407 (6)	0.0820 (13)	
H11A	0.1767	0.8585	1.1205	0.098*	
C12	0.3433 (4)	0.8850 (2)	1.1137 (6)	0.0664 (11)	
C13	0.3544 (4)	0.9064 (2)	1.2683 (6)	0.0800 (13)	
H13A	0.2924	0.9042	1.3273	0.096*	
C14	0.4564 (6)	0.9312 (3)	1.3369 (7)	0.0982 (17)	
H14A	0.4638	0.9446	1.4427	0.118*	
C15	0.5474 (5)	0.9361 (3)	1.2496 (9)	0.0971 (17)	
H15A	0.6165	0.9531	1.2958	0.116*	
C16	0.5364 (4)	0.9164 (3)	1.0977 (8)	0.0981 (17)	
H16A	0.5981	0.9207	1.0385	0.118*	
C17	0.4361 (4)	0.8899 (3)	1.0275 (6)	0.0888 (14)	
H17A	0.4304	0.8754	0.9223	0.107*	

### Atomic displacement parameters (Å^2^)

**Table:**

	*U*^11^	*U*^22^	*U*^33^	*U*^12^	*U*^13^	*U*^23^
Br1	0.0582 (3)	0.0681 (3)	0.1219 (5)	−0.0028 (2)	0.0096 (3)	−0.0066 (3)
Br2	0.1595 (6)	0.0592 (3)	0.1144 (5)	−0.0146 (3)	−0.0012 (4)	−0.0116 (3)
O1	0.106 (3)	0.087 (2)	0.071 (2)	−0.0081 (19)	0.022 (2)	0.0099 (17)
O2	0.092 (2)	0.086 (2)	0.095 (3)	−0.0176 (18)	0.042 (2)	0.0054 (18)
O3	0.085 (2)	0.131 (3)	0.068 (2)	−0.051 (2)	0.0126 (19)	0.005 (2)
N1	0.075 (3)	0.057 (2)	0.063 (3)	−0.0025 (18)	0.008 (2)	−0.0008 (18)
N2	0.096 (3)	0.091 (3)	0.069 (3)	−0.004 (2)	0.005 (3)	0.007 (2)
C1	0.076 (3)	0.061 (3)	0.109 (4)	−0.003 (2)	−0.005 (3)	0.001 (3)
C2	0.080 (4)	0.069 (3)	0.129 (5)	−0.020 (3)	−0.011 (3)	0.010 (3)
C3	0.080 (4)	0.098 (4)	0.108 (4)	−0.023 (3)	0.003 (3)	0.007 (3)
C4	0.086 (4)	0.091 (4)	0.125 (5)	0.015 (3)	0.024 (3)	0.004 (3)
C5	0.083 (3)	0.065 (3)	0.101 (4)	0.001 (3)	0.013 (3)	0.006 (3)
C6	0.065 (3)	0.061 (3)	0.065 (3)	−0.006 (2)	0.000 (2)	0.000 (2)
C7	0.088 (4)	0.055 (3)	0.076 (4)	0.002 (2)	0.021 (3)	−0.002 (2)
C8	0.067 (3)	0.064 (2)	0.055 (3)	−0.009 (2)	0.014 (3)	0.000 (2)
C9	0.063 (3)	0.074 (3)	0.072 (4)	−0.022 (2)	0.012 (3)	−0.008 (2)
C10	0.066 (3)	0.087 (3)	0.079 (3)	−0.014 (2)	0.017 (3)	0.004 (3)
C11	0.082 (3)	0.083 (3)	0.083 (4)	−0.019 (3)	0.015 (3)	0.000 (3)
C12	0.067 (3)	0.068 (3)	0.064 (3)	−0.005 (2)	0.009 (3)	0.000 (2)
C13	0.094 (4)	0.067 (3)	0.080 (4)	−0.010 (2)	0.017 (3)	−0.011 (2)
C14	0.127 (5)	0.081 (3)	0.084 (4)	−0.012 (3)	−0.006 (4)	−0.012 (3)
C15	0.077 (4)	0.078 (3)	0.130 (6)	−0.007 (3)	−0.025 (4)	0.003 (3)
C16	0.061 (3)	0.116 (4)	0.116 (5)	0.003 (3)	0.001 (4)	0.017 (4)
C17	0.070 (3)	0.115 (4)	0.082 (4)	0.009 (3)	0.014 (3)	0.006 (3)

### Geometric parameters (Å, °)

**Table:**

Br1—C10	2.008 (4)	C5—H5A	0.9300
Br2—C11	1.990 (5)	C7—C8	1.422 (6)
O1—N2	1.354 (5)	C8—C9	1.442 (6)
O1—C7	1.411 (5)	C9—C10	1.533 (6)
O2—C7	1.203 (5)	C10—C11	1.455 (6)
O3—C9	1.206 (5)	C10—H10A	0.9800
N1—N2	1.304 (5)	C11—C12	1.505 (6)
N1—C8	1.351 (5)	C11—H11A	0.9800
N1—C6	1.448 (5)	C12—C13	1.367 (6)
C1—C6	1.361 (5)	C12—C17	1.382 (6)
C1—C2	1.374 (6)	C13—C14	1.372 (7)
C1—H1A	0.9300	C13—H13A	0.9300
C2—C3	1.361 (6)	C14—C15	1.370 (7)
C2—H2A	0.9300	C14—H14A	0.9300
C3—C4	1.369 (6)	C15—C16	1.338 (7)
C3—H3A	0.9300	C15—H15A	0.9300
C4—C5	1.376 (6)	C16—C17	1.370 (7)
C4—H4A	0.9300	C16—H16A	0.9300
C5—C6	1.365 (5)	C17—H17A	0.9300
			
N2—O1—C7	111.2 (4)	C8—C9—C10	114.9 (4)
N2—N1—C8	114.6 (4)	C11—C10—C9	115.6 (4)
N2—N1—C6	116.6 (4)	C11—C10—Br1	108.0 (3)
C8—N1—C6	128.8 (4)	C9—C10—Br1	103.6 (3)
N1—N2—O1	105.3 (4)	C11—C10—H10A	109.8
C6—C1—C2	119.2 (4)	C9—C10—H10A	109.8
C6—C1—H1A	120.4	Br1—C10—H10A	109.8
C2—C1—H1A	120.4	C10—C11—C12	116.1 (4)
C3—C2—C1	120.1 (5)	C10—C11—Br2	104.4 (3)
C3—C2—H2A	119.9	C12—C11—Br2	109.5 (3)
C1—C2—H2A	119.9	C10—C11—H11A	108.8
C2—C3—C4	119.9 (5)	C12—C11—H11A	108.8
C2—C3—H3A	120.1	Br2—C11—H11A	108.8
C4—C3—H3A	120.1	C13—C12—C17	118.8 (4)
C3—C4—C5	120.9 (5)	C13—C12—C11	119.8 (4)
C3—C4—H4A	119.5	C17—C12—C11	121.4 (4)
C5—C4—H4A	119.5	C12—C13—C14	120.4 (5)
C6—C5—C4	118.0 (4)	C12—C13—H13A	119.8
C6—C5—H5A	121.0	C14—C13—H13A	119.8
C4—C5—H5A	121.0	C15—C14—C13	120.0 (5)
C1—C6—C5	122.0 (4)	C15—C14—H14A	120.0
C1—C6—N1	119.8 (4)	C13—C14—H14A	120.0
C5—C6—N1	118.2 (4)	C16—C15—C14	119.7 (5)
O2—C7—O1	119.7 (5)	C16—C15—H15A	120.2
O2—C7—C8	136.8 (5)	C14—C15—H15A	120.2
O1—C7—C8	103.5 (4)	C15—C16—C17	121.3 (5)
N1—C8—C7	105.5 (4)	C15—C16—H16A	119.3
N1—C8—C9	125.7 (4)	C17—C16—H16A	119.3
C7—C8—C9	128.7 (4)	C16—C17—C12	119.7 (5)
O3—C9—C8	124.2 (4)	C16—C17—H17A	120.2
O3—C9—C10	120.9 (4)	C12—C17—H17A	120.2
			
C8—N1—N2—O1	−0.1 (5)	N1—C8—C9—O3	−1.4 (7)
C6—N1—N2—O1	178.4 (3)	C7—C8—C9—O3	176.6 (4)
C7—O1—N2—N1	0.1 (4)	N1—C8—C9—C10	−179.0 (4)
C6—C1—C2—C3	0.3 (8)	C7—C8—C9—C10	−1.1 (6)
C1—C2—C3—C4	−0.2 (8)	O3—C9—C10—C11	29.5 (6)
C2—C3—C4—C5	0.0 (8)	C8—C9—C10—C11	−152.7 (4)
C3—C4—C5—C6	0.0 (8)	O3—C9—C10—Br1	−88.4 (4)
C2—C1—C6—C5	−0.3 (7)	C8—C9—C10—Br1	89.4 (4)
C2—C1—C6—N1	−179.3 (4)	C9—C10—C11—C12	−176.7 (4)
C4—C5—C6—C1	0.1 (7)	Br1—C10—C11—C12	−61.3 (5)
C4—C5—C6—N1	179.1 (4)	C9—C10—C11—Br2	62.6 (4)
N2—N1—C6—C1	106.8 (5)	Br1—C10—C11—Br2	178.07 (17)
C8—N1—C6—C1	−74.9 (6)	C10—C11—C12—C13	123.0 (5)
N2—N1—C6—C5	−72.2 (5)	Br2—C11—C12—C13	−119.1 (4)
C8—N1—C6—C5	106.1 (5)	C10—C11—C12—C17	−56.4 (6)
N2—O1—C7—O2	−179.6 (4)	Br2—C11—C12—C17	61.6 (5)
N2—O1—C7—C8	−0.1 (4)	C17—C12—C13—C14	−1.1 (7)
N2—N1—C8—C7	0.0 (5)	C11—C12—C13—C14	179.6 (4)
C6—N1—C8—C7	−178.3 (3)	C12—C13—C14—C15	1.4 (7)
N2—N1—C8—C9	178.3 (4)	C13—C14—C15—C16	−0.3 (8)
C6—N1—C8—C9	0.1 (6)	C14—C15—C16—C17	−1.1 (8)
O2—C7—C8—N1	179.3 (5)	C15—C16—C17—C12	1.4 (8)
O1—C7—C8—N1	0.1 (4)	C13—C12—C17—C16	−0.3 (7)
O2—C7—C8—C9	1.0 (8)	C11—C12—C17—C16	179.0 (4)
O1—C7—C8—C9	−178.2 (4)		

### Hydrogen-bond geometry (Å, °)

**Table:**

*D*—H···*A*	*D*—H	H···*A*	*D*···*A*	*D*—H···*A*
C10—H10A···O2	0.98	2.35	3.028 (6)	126
C13—H13A···O2^i^	0.93	2.49	3.312 (6)	147

Symmetry codes: (i) *x*, *y*, *z*+1.
